# The incidence of post-traumatic stress disorder among survivors after earthquakes:a systematic review and meta-analysis

**DOI:** 10.1186/s12888-016-0891-9

**Published:** 2016-06-07

**Authors:** Wenjie Dai, Long Chen, Zhiwei Lai, Yan Li, Jieru Wang, Aizhong Liu

**Affiliations:** Department of Epidemiology and Health Statistics, School of Xiangya Public Health, Central South University, Changsha, China; Zhuhai Center for Disease Control and Prevention, Zhuhai, China; Department of Pediatrics, University of Pittsburgh School of Medicine, Pittsburgh, USA

**Keywords:** Post-traumatic stress disorder, Earthquake, Incidence, Systematic review, Meta-analysis

## Abstract

**Background:**

Post-traumatic stress disorder (PTSD) is a common psychological disorder caused by unusual threats or catastrophic events. Little is known about the combined incidence of PTSD after earthquakes. This study aimed at evaluating the combined incidence of PTSD among survivors after earthquakes using systematic review and meta-analysis.

**Methods:**

The electronic databases of PubMed, Embase, Web of Science and PsycARTICLES were searched for relevant articles in this study. Loney criteria were used to assess the quality of eligible articles. The combined incidence of PTSD was estimated by using the Freeman-Tukey double arcsine transformation method. Subgroup analyses were conducted using the following variables: the time of PTSD assessment, gender, educational level, marital status, damage to one’s house, bereavement, injury of body and witnessing death.

**Results:**

Forty-six eligible articles containing 76,101 earthquake survivors met the inclusion criteria, of which 17,706 were diagnosed as having PTSD. Using a random effects model, the combined incidence of PTSD after earthquakes was 23.66 %. Moreover, the combined incidence of PTSD among survivors who were diagnosed at not more than 9 months after earthquake was 28.76 %, while for survivors who were diagnosed at over nine months after earthquake the combined incidence was 19.48 %. A high degree of heterogeneity (I^2^ = 99.5 %, p<0.001) was observed in the results, with incidence ranging from 1.20 to 82.64 %. The subgroup analyses showed that the incidence of PTSD after earthquake varied significantly across studies in relation to the time of PTSD assessment, gender, educational level, damage to one’s house, bereavement, injury of body and witnessing death. However, stratified analyses could not entirely explain the heterogeneity in the results.

**Conclusions:**

Given the high heterogeneity observed in this study, future studies should aim at exploring more possible risk factors for PTSD after earthquakes, especially genetic factors. In spite of that, the results of this study suggest that nearly 1 in 4 earthquake survivors are diagnosed as having PTSD. Therefore, the local government should plan effective psychological interventions for earthquake survivors.

**Electronic supplementary material:**

The online version of this article (doi:10.1186/s12888-016-0891-9) contains supplementary material, which is available to authorized users.

## Background

Earthquakes are one of the most destructive and frequently occurring natural disasters [1]. They often strike unexpectedly without warning and bring adverse impact to a great deal of people [2]. Earthquakes have caused a lot of deaths and injuries throughout the human history, leaving survivors with endless panic and some mental problems, including post-traumatic stress disorder (PTSD) [3].

PTSD is a psychological disorder caused by unusual threats or catastrophic events. It has been regarded as the most prevalent type of psychiatric disorder after disasters [4], including earthquake, tsunami, flood, etc. Numerous studies have reported the estimated incidence of probable PTSD or PTSD symptoms among earthquake survivors. However, an enormous disparity does exist in the reported incidence of PTSD symptoms.

Previous studies have shown that the estimated incidence of PTSD among earthquake survivors varied from 1.20 [5] to 82.64 % [6]. This variation might have been associated with factors such as the variation in the intensity of the earthquakes, the variation in the degree victims were exposed to the catastrophe, the variation in the assessment time of PTSD after the trauma emerged, the variation in the quantity of property lost and whether bereavement occurred or not [7–9].

Improving the understanding of the accuracy of the incidence of PTSD after earthquakes is important as it may draw more public attention which would lead into finding some effective psychological interventions. However, there has been no systematic review attempting to synthesize these data until now. In this study, a systematic review and meta-analysis of previously published articles on the incidence of PTSD among earthquake survivors were performed in order to obtain a combined incidence of PTSD after earthquakes.

## Methods

### Search strategy

This systematic review was conducted under the guidance of Preferred Reporting Items for Systematic Reviews and Meta-Analyses (PRISMA) criteria and literature searches were conducted on December 14, 2015. The electronic databases of PubMed, Embase, Web of Science and PsycARTICLES were searched for relevant articles from their inceptions to the present. Search terms for PubMed were:"Earthquakes"[Mesh] AND "Stress Disorders, Post-Traumatic"[Mesh]. Search terms for Embase were: ('post traumatic stress disorder': ab,ti OR 'posttraumatic stress disorder':ab,ti OR 'PTSD':ab,ti) AND ('earthquake':ab,ti' OR earthquakes':ab,ti). These terms were adapted for the other databases and the detailed search strategies are shown in the Additional file 1. The reference list of each published article was also examined to identify relevant studies.

### Eligibility criteria

Studies eligible for this review had to fulfill the following inclusion criteria: (1) studies must have been observational and must have assessed PTSD with specific reference to the earthquake; (2) studies must have examined PTSD diagnosis at least 1 month after the earthquake; (3) studies must have identified PTSD by established psychiatric interviews according to the Diagnostic and Statistical Manual of Mental Disorders, 4th edition (DSM-IV) criteria or the self-reporting questionnaires that based on DSM-IV; (4) the total sample size of each study must have been no less than 300; (5) the incidence of PTSD among survivors after earthquakes had to be provided or could be calculated from the data the articles provided. The exclusion criteria were: (1) articles were not written in English; (2) articles were reviews, reports, comments or book chapters; (3) erroneous or contradictory information was included in the articles; (4) any kinds of interventions were included in the articles or the participants of the studies were special, such as firefighters, doctors, etc. Besides, the samples in the study should not overlap with other identified studies with the same follow-up period. If two or more publications with the same follow-up period shared all the data or data subsets then only one publication with the largest sample size was included; if the sample sizes of similar studies were the same, then the earlier publication was included; if the data or data subsets were from duplicate publications but they had different follow-up time, then all of them were included.

### Data abstraction

Data abstraction was conducted independently by two investigators and any discrepancy between them was resolved by consensus. For the purpose of the meta-analysis, data retrieved from literature included: (1) the title of the study, the first author, the year of publication, the geographic area of the study, the time of PTSD assessment and the quality of the literature; (2) the diagnostic tool of PTSD, the number of victims with PTSD, the number of final participants of a survey, the incidence of PTSD, the demographic information of the participants (age, gender, nationality, religious beliefs, marital status, educational level) and the intensity of the earthquakes measured by witnessing death or not, house damage or not, injury or not and bereavement or not. All the information was collected by EpiData 3.0.

### Quality evaluation

The quality of eligible articles was assessed by using the evaluation criteria for prevalence or incidence studies as proposed and recommended by Loney [10]. The evaluation criteria consist of eight items namely, (1) participants (random sample or population); (2) the description of study procedure; (3) adequate sample size (≥300); (4) efficient diagnostic tools; (5) unbiased appraisal of the outcome; (6) adequate response rate; (7) subgroup analysis; and (8) the detailed description of participants. An article scores points equal to the number of items it has satisfied and if the article satisfies one item of the criteria, it will be given 1 point. Thus, the total quality scores of articles range from 0 to 8 points.

### Statistical analysis

The number of PTSD victims and the total sample size were extracted from the original literature for the calculation of incidence. Data were analyzed using the statistical software R version 3.2.0. Freeman-Tukey transformation of inverse hyperbolic sine function was used to calculate the combined incidence. Heterogeneity was evaluated both visually by means of forest-plots and using the χ^2^ test on Cochrane’s Q statistic, and it was then quantified by calculating the I^2^. Heterogeneity test was considered statistically significant when *p* ≤ 0.05. In this case the data were analyzed using a random effects model. In contrast, if *p*>0.05, a fixed effects model was used to analyze the data.

Subgroup analyses were carried out to identify the source of heterogeneity in the following variables: the time of PTSD assessment, gender, educational level, marital status, damage to one’s house, bereavement, injury of body and witnessing death. A comparison of the incidence between subgroups was done by carrying out a χ^2^ test using the software, Statistical Package for the Social Sciences (SPSS) version 19.0. Sensitivity analysis was carried out to verify the influence of low-quality studies on the stability of the combined incidence. In order to verify whether publication bias might have an influence on the validity of the incidence, linear regression method was used and an Egger funnel plot was then presented. All p values were two sided and the cut-off for statistical significance was set at 0.05.

## Results

### Literature search

**Fig. 1:**
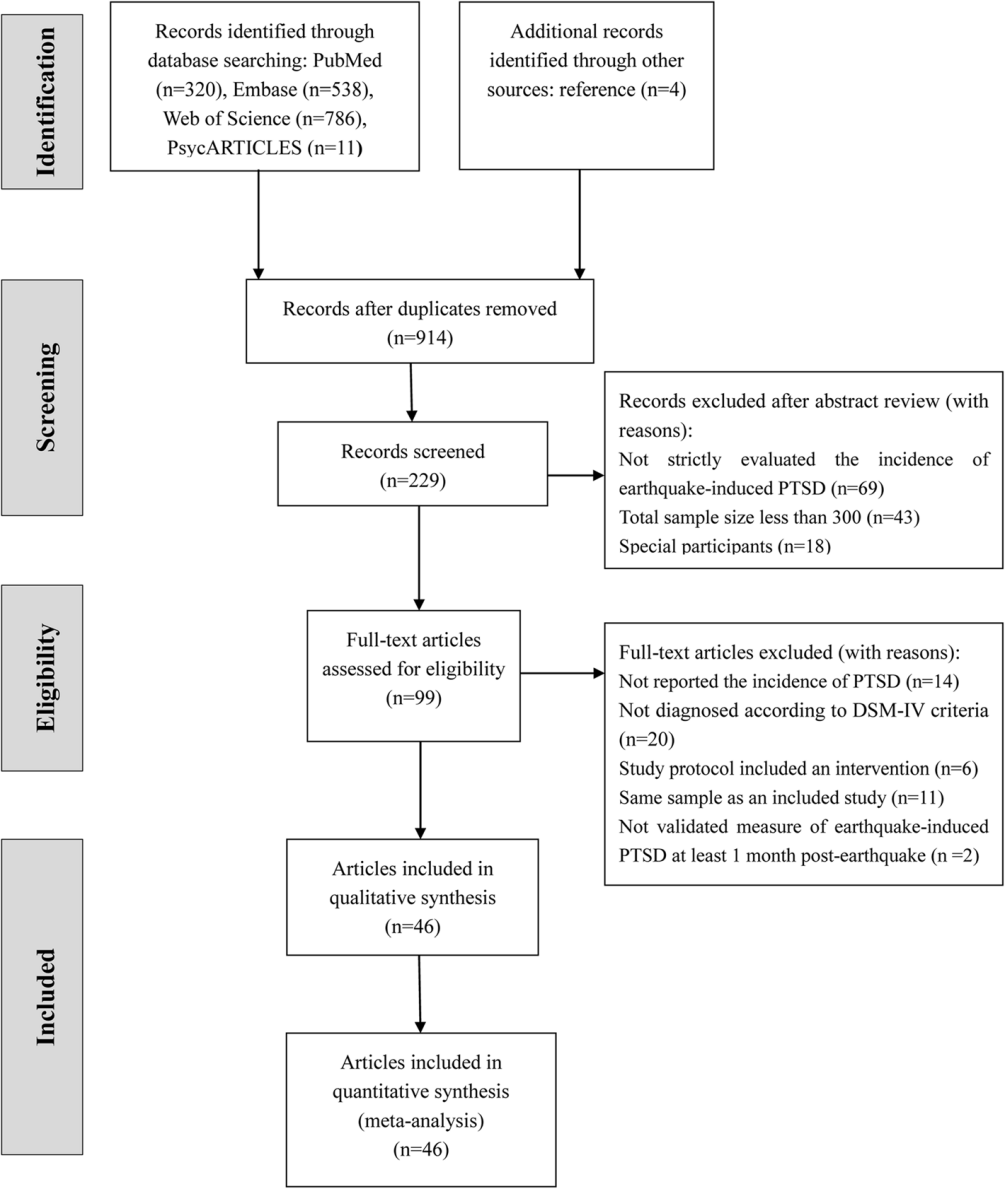
PRISMA flow chart of article selection; illustration of how eligible articles were selected

An aggregate of 1,659 relevant articles were identified for this study, of which 99 full papers were shortlisted for eligibility test. Further examination of the 99 full papers resulted in 14 articles excluded for not reporting the incidence of PTSD; 20 articles excluded for identifying PTSD neither by established psychiatric interviews according to the DSM-IV criteria nor by the self-reporting questionnaires that based on DSM-IV; 6 articles excluded for including interventions; 2 articles excluded for not measuring earthquake-induced PTSD at least 1 month post-earthquake and 11 articles excluded for repeated data with same follow-up periods. Thus, 46 eligible articles were finally included in this study (Fig. 1).

### Characteristics of eligible articles

The 46 eligible articles considered destructive earthquakes of magnitudes ranging from 4.3 to 9.0 on a Richter scale, which occurred between 1999 and 2013. They analyzed and described the PTSD of the survivors of these catastrophes with follow-up periods ranging from 1 to 60 months. Only 6 of the 46 eligible articles analyzed and described longitudinal studies while the rest analyzed and described cross-sectional studies. In addition, 40 eligible articles identified PTSD only by self-reporting questionnaires and the other 6 eligible articles identified PTSD through clinical interviews. In the quality assessment of the 46 eligible articles, 9 articles scored 7 points; 15 articles scored 6 points; 18 articles scored 5 points and 4 articles scored 4 points. The characteristics of the 46 eligible articles are summarized in Table 1.

**Table 1:** Characteristics of the studies included in this systematic review and meta-analysis

Author	Year	Study design	Region	Richer scale	Questionnaire	Clinical interview	Time after earthquake (month)	Victims with PTSD	Total sample size	Quality evaluation
Wu et al [22]	2006	Cross-sectional	Chi-Chi,Taiwan	7.3	NO	MINI	36	18	405	5
Chou et al [23]	2005	Cross-sectional	Chi-Chi,Taiwan	7.3	NO	MINI	6	35	442	6
Flores et al [24]	2014	Cross-sectional	Pisco, Peru	7.9	PCL-C	NO	48	161	1012	6
Kadak et al [25]	2013	Cross-sectional	Van, Turkey	7.2	CPTSD-RI	NO	6	295	725	5
Zhou et al [26]	2015	Cross-sectional	Wenchuan, China	8.0	PCL-C	NO	12	224	817	5
Emin et al [27]	2006	Cross-sectional	Marma, Turkey	7.4	TSSC	NO	36	131	683	5
Metin et al [28]	2004	Cross-sectional	Marma, Turkey	7.4	TSSC	NO	14	177	950	6
Zhang et al [29]	2015	Cross-sectional	Wenchuan, China	8.0	PCL-C	NO	60	63	684	6
Peng et al [30]	2009	Cross-sectional	Wenchuan, China	8.0	HTQ	NO	2.5	251	447	7
Roussos et al [31]	2005	Cross-sectional	Ano Liosia, Greece	5.9	PTSD-RI	NO	3	87	1937	4
Jude et al [32]	2015	Cross-sectional	Haiti	7.0	IES-R	NO	30	322	872	5
Fu et al [33]	2013	Cross-sectional	Wenchuan, China	8.0	PCL-C	NO	12	420	2987	5
Hsu et al [34]	2002	Cross-sectional	Chi-Chi,Taiwan	7.3	NO	ChIPS	1.5	70	323	7
Jia et al [35]	2015	Cross-sectional	Wenchuan, China	8.0	CPSS	NO	12	179	631	5
Tian et al [36]	2014	Cross-sectional	Wenchuan, China	8.0	PCL-C	SCID	36	261	4604	6
Wang et al [37]	2013	Cross-sectional	Yingjiang, China	5.8	CPSS	NO	1	445	1198	4
Zhang et al [38]	2015	Longitudinal study	Lushan, China	7.0	CRIES	NO	3	834	2229	5
							6	556	2299	
Cem et al [39]	2013	Cross-sectional	Konya, Turkey	4.3	CPTSD-RI	NO	6	110	450	7
Chan et al [40]	2011	Cross-sectional	Wenchuan, China	8.0	IES-R	NO	7.5	526	1725	5
Fan et al [41]	2011	Cross-sectional	Wenchuan, China	8.0	PTSD-SS	NO	6	329	2081	6
Guo et al [42]	2014	Longitudinal study	Wenchuan, China	8.0	IES-R	NO	2	620	1066	6
							8	297	1344	
							14	239	1210	
							26	223	1174	
							44	102	1281	
Jia et al [43]	2013	Longitudinal study	Wenchuan, China	8.0	CPTSD-RI	NO	15	74	596	7
							36	46	430	
Ying et al [44]	2013	cross-sectional	Wenchuan, China	8.0	CPSS	NO	12	262	3052	5
Xu et al [45]	2011	cross-sectional	Wenchuan, China	8.0	PCL	NO	12	835	2080	6
Ali et al [46]	2012	cross-sectional	Kashmir, Pakistan	7.6	DTS	NO	30	124	300	5
Ayub et al [47]	2012	cross-sectional	Kashmir, Pakistan	7.6	CRIES	NO	18	699	1078	6
Jude et al [48]	2014	cross-sectional	Haiti	7.0	IES-R	NO	30	498	1355	6
Gigantesco et al [49]	2013	cross-sectional	L’Aquila, Italy	6.3	NO	Mini	12	39	957	7
Liu et al [50]	2010	Longitudinal study	Wenchuan, China	8.0	PCL-C	NO	4	165	1474	6
							6	129	1474	
							9	100	1474	
							12	84	1474	
Naeem et al [51]	2011	cross-sectional	Kashmir, Pakistan	7.6	TSSC	NO	18	601	1291	7
Parvaresh et al [52]	2009	cross-sectional	Bam,Iran	6.3	NO	Watson interview	4	182	433	5
Takeda et al [53]	2013	cross-sectional	Great East Japan	9.0	IES-R	NO	9	118	1180	5
Wang et al [54]	2011	cross-sectional	Wenchuan, China	8.0	PTSD-SS	NO	1	257	409	7
Wang et al [55]	2013	cross-sectional	Wenchuan, China	8.0	PCL-C	NO	42	145	319	6
Wang et al [56]	2012	cross-sectional	Wenchuan, China	8.0	CRIES	NO	10	522	1841	7
Wen et al [57]	2012	cross-sectional	Wenchuan, China	8.0	PCL-C	NO	36	113	2525	6
Yuqing et al [6]	2011	cross-sectional	Wenchuan, China	8.0	IES-R	NO	2	790	956	4
Zhang et al [5]	2012	Longitudinal study	Wenchuan, China	8.0	PCL-C	NO	6	53	548	5
							12	7	584	
							18	9	548	
Zhang et al [58]	2011	cross-sectional	Wenchuan, China	8.0	PCL-C	NO	12	311	1181	6
Zhen et al [59]	2012	cross-sectional	Yushu, China	7.1	PCL-C	NO	3	170	505	5
Hou et al [60]	2011	Longitudinal study	Wenchuan, China	8.0	PCL-C	NO	3	613	1677	5
							6	515	1677	
							9	416	1677	
							12	373	1677	
Lau et al [61]	2010	cross-sectional	Wenchuan, China	8.0	CRIES	NO	1	741	3324	4
Liu et al [62]	2010	cross-sectional	Wenchuan, China	8.0	PCL-C	NO	9	346	569	6
Ying et al [63]	2014	cross-sectional	Wenchuan, China	8.0	CPSS	NO	12	101	788	5
Kun et al [64]	2013	cross-sectional	Wenchuan, China	8.0	HTQ	NO	3	529	1820	7
Sezgin et al [65]	2012	cross-sectional	South Eastern Turkey	6.4	PDS	NO	12	764	1253	5

*MINI* mini international neuropsychiatric interview, *PCL-C* PTSD checklist-civilian version, *CPTSD-RI* child PTSD–reaction index, *TSSC* traumatic stress symptom checklist, *HTQ* harvard trauma questionnaire, *PTSD-RI* PTSD reaction index, *IES-R* impact of event scale-revised, *ChIPS* children’s interview for psychiatric syndromes, *CPSS* child PTSD symptom scale, *SCID* structured clinical interview for DSM-IV disorders, *CRIES* children's revised impact of event scale, *PTSD-SS* PTSD self-rating scale, *DTS* Davidson trauma scale, *PCL* PTSD checklist, *PDS* post traumatic stress diagnostic scale

### Combined incidence of PTSD after earthquakes

**Fig. 2:**
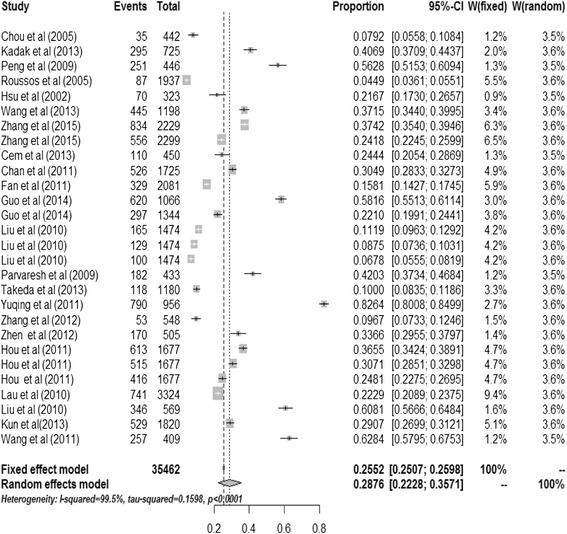
Incidence tree of PTSD diagnosed at not more than nine months follow-up after earthquake; graphical representation of a meta-analysis of incidence of PTSD diagnosed at not more than nine months follow-up after earthquake

**Fig. 3:**
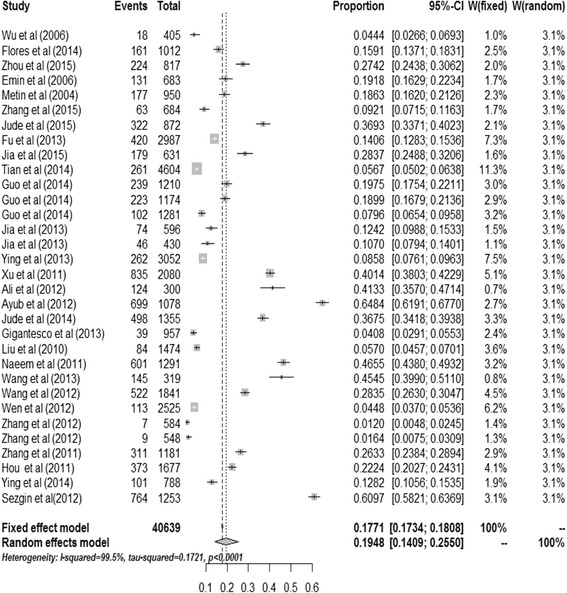
Incidence tree of PTSD diagnosed at over nine months follow-up after earthquake; graphical representation of a meta-analysis of incidence of PTSD diagnosed at over nine months follow-up after earthquake

A total of 76,101 survivors after earthquakes were available for this systematic review and meta-analysis, of which 17,706 victims were identified to have PTSD. The incidence of PTSD among survivors after earthquakes ranged from 1.20 [5] to 82.64 % [6] and the heterogeneity test of the included studies showed that they were heterogeneous (I^2^ = 99.5 %; *p*<0.001). Therefore, the random effects model was used to assess the combined incidence of PTSD. The combined incidence of PTSD among survivors after earthquakes was 23.66 % (95 % confidence interval (95 % CI): 19.34-28.27 %). The combined incidence of PTSD among survivors who were diagnosed at not more than 9 months after earthquake was 28.76 % (95 % CI: 22.28-35.71 %), while for survivors who were diagnosed at over nine months after earthquake the combined incidence was 19.48 % (95 % CI:14.09-25.50 %). Figures 2 and 3 show the details.

### Subgroup analyses

Subgroup analyses were performed with respect to the time of PTSD assessment after earthquakes, gender, educational level, marital status, damage to one’s house, bereavement, injury of body, and witnessing death (Table 2). The results indicated that studies with longer follow-up periods (>9 months) showed lower incidence of PTSD (combined incidence = 19.48 %, 95 % CI = 14.09-25.50 %) than did studies with shorter follow-up periods (≤9 months; combined incidence = 28.76 %, 95 % CI = 22.28-35.71 %). The combined incidence of PTSD among female survivors after earthquakes (34.82 %, 95 % CI: 26.85-43.24 %) was higher than that of male survivors (22.57 %, 95 % CI: 16.53-29.23 %). Besides, the combined incidence of PTSD among survivors after earthquakes with educational level at most elementary school (31.56 %, 95 % CI: 21.22-42.90 %) was higher than that of survivors with educational level higher than elementary school (19.76 %, 95 % CI: 14.33-25.82 %). Furthermore, the combined incidence of PTSD among survivors who had their houses damaged (38.49 %, 95 % CI: 25.11-52.82 %) was higher than that of survivors with their houses not damaged (23.97 %, 95 % CI: 8.08-44.81 %). In addition, the combined incidence of PTSD among survivors with bereavement after earthquake (39.10 %, 95 % CI: 25.74-53.33 %) was higher than that of survivors without bereavement (19.92 %, 95 % CI: 10.89-30.83 %). Also, the combined incidence of PTSD among injured survivors after earthquake (23.28 %, 95 % CI: 13.91-34.16 %) was higher than that of non-injured survivors (9.63 %, 95 % CI: 3.62-18.09 %). What is more, the combined incidence of PTSD among survivors who had witnessed death after earthquakes (26.28 %, 95 % CI: 7.05-52.14 %) was higher than that of survivors who had not witnessed death (14.69 %, 95 % CI: 0.06-41.35 %). However, stratification according to these parameters could not entirely explain the heterogeneity of the results, with I^2^ still being high within each stratum.

**Table 2:** Subgroup analyses of the incidence of PTSD after earthquakes^a^Incidence rates were obtained using a random-effects model

Group	Number of studies	Incidence^a^ (95 % CI)%	*p* value (heterogeneity**)	I^2^(%)	*p* value (interaction***)
Total	60	23.66 (19.34–28.27)	<0.001	99.5	
Assessment time after earthquake					<0.001
≤9 months	28	28.76 (22.28–35.71)	<0.001	99.5	
>9 months	32	19.48 (14.09–25.50)	<0.001	99.5	
Gender					<0.001
Male	26	22.57 (16.53–29.23)	<0.001	98.8	
Female	29	34.82 (26.85–43.24)	<0.001	99.3	
Educational level					<0.001
Elementary school or below	13	31.56 (21.22–42.90)	<0.001	99.1	
Beyond elementary school	29	19.76 (14.33–25.82)	<0.001	99.3	
Marital status					0.069
Married	7	25.61 (13.74–439.68)	<0.001	99.0	
Unmarried	7	22.74(12.23–35.32)	<0.001	97.2	
Damage to one’s house					<0.001
Yes	6	38.49 (25.11–52.82)	<0.001	98.2	
No	6	23.97 (8.08–44.81)	<0.001	99.3	
Bereavement					<0.001
Yes	12	39.10 (25.74–53.33)	<0.001	98.5	
No	10	19.92 (10.89–30.83)	<0.001	99.2	
Injury of body					<0.001
Yes	6	23.28 (13.91–34.16)	<0.001	96.4	
No	5	9.63 (3.62–18.09)	<0.001	98.2	
Witnessed death					<0.001
Yes	3	26.28 (7.05–52.14)	<0.001	98.8	
No	3	14.69 (0.06–41.35)	<0.001	99.3	

** *p* values for heterogeneity across studies were computed using Cochrane’s Q test

*** *p* values for comparisons between subgroups were computed using the χ^2^ test with one degree of freedom

### Sensitivity and bias analysis

After excluding articles with the quality evaluation score equal to 4 points for this meta-analysis, the combined incidence of PTSD declined from 23.66 to 22.95 %. This small change in combined incidence of PTSD, after excluding low quality eligible articles, indicated low sensitivity and hence credible results of this study.

**Fig. 4:**
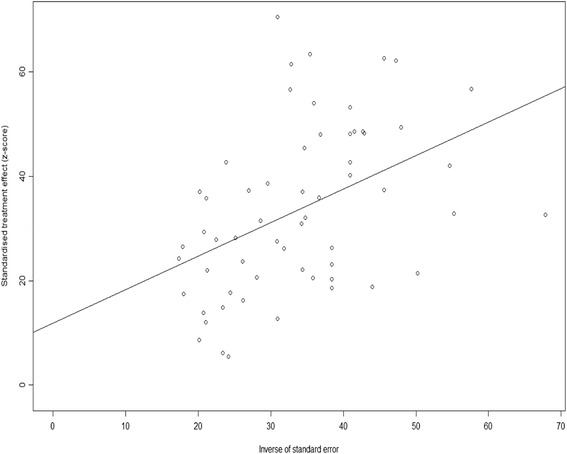
Egger plot of literatures on the incidence of PTSD after earthquakes. Egger plot/graph for assessing publication bias

Publication bias was assessed by using the linear regression analysis. An Egger funnel plot was produced and it indicated that there was a negligible chance for publication bias (Fig. 4). In agreement with the Egger funnel plot, Egger’s test scored a p value of 0.057, implying that there was a very low probability of publication bias.

## Discussion

Literature search for this meta-analysis found no evidence of existing meta-analyses that investigated the incidence of PTSD among survivors after earthquakes. Therefore, this is probably the first meta-analysis to investigate the incidence of PTSD among survivors after earthquakes. This meta-analysis considered articles which analyzed and described PTSD among earthquake survivors, which happened between 1999 and 2013 all over the world, whose magnitude on Richter scale ranged from 4.3 to 9.0. It is therefore understood that the results of this meta-analysis could, to some extent, reflect the actual and precise incidence of PTSD after earthquakes in the world. The 46 eligible articles for this meta-analysis accounted for 76,101 earthquake survivors, of which 17,706 had been diagnosed with PTSD. It was found, from this information, that the combined incidence of PTSD among survivors after earthquakes was 23.66 % (95 % CI: 19.34-28.27 %). Edmondson D [11] showed that the prevalence of PTSD in survivors of stroke and transient ischemic attack was 13 % (95 % CI: 11 %-16 %) and Chen L [12] found that the incidence of PTSD after floods was 15.74 % (95 % CI: 11.25 %-20.82 %). Thus, this study’s combined incidence of PTSD among earthquake survivors was much higher than that found among flood survivors and stroke survivors. This was mainly because earthquakes were often much more devastating and destructive, and often happened unexpectedly without warning. Therefore they might have brought more damage to one’s properties and health, including both physical health and mental health [13]. Hence, the local government should pay more attention to the mental health of earthquake survivors and try to find some effective interventions to provide high standard rehabilitation services.

The subgroup analyses showed that the combined incidence of PTSD among survivors who were identified at not more than nine months after earthquakes was 28.76 %, while for survivors who were assessed of PTSD at over nine months after the earthquakes the combined incidence of PTSD was 19.48 %. This variation tendency in the incidence of PTSD was consistent with Edmondson D’s study [11]. The incidence of PTSD symptoms were higher in the immediate aftermath of the earthquake [14]. In line with some previous studies [15], the subgroup analyses also indicated that damage to one’s house, bereavement, injury of body and witnessing death would contribute to the different incidences of PTSD, suggesting that those who suffered more property loss or personal injury or had witnessed death or had experienced bereavement were more likely to develop PTSD [16]. In addition, the subgroup analyses showed that gender and educational level may lead to different incidences of PTSD after earthquakes. Females and those who had low educational level were more likely to develop PTSD. Those findings were consistent with conclusions of many studies in disaster psychology [17, 18]. Some studies revealed that women and people with lower educational level were less likely to use positive coping strategies, were more sensitive to threats and tended to interpret disasters more negatively [19, 20].

In this meta-analysis, substantial information was obtained for determining the combined incidence. However, quality assessment showed that most of the eligible articles did not report the 95 % CI of the observed incidence and lacked enough subgroup analyses. In addition, they identified PTSD by self-reporting questionnaires rather than clinical interviews by professional psychiatrists, as a consequence of which, the combined incidence of PTSD may have been overestimated. Furthermore, subgroup analyses did not identify major sources of the heterogeneity although a high degree of heterogeneity between studies was observed. Therefore, there might be a considerable amount of uncertainty regarding the combined incidence of PTSD after earthquakes. It is also believed that genetic background might have played an important role in the incidence of PTSD after earthquakes with increasing evidence showing that genetic factors and gene-environment interaction were both associated with the onset of PTSD [21]. Future research should, therefore, explore more potential risk factors for PTSD after earthquakes, especially genetic background.

Also, this study did not observe significant publication bias and the sensitivity was low after excluding articles with the quality evaluation score equal to 4. The strengths of this study included its large sample size and a large number of subgroup analyses. However, several limitations do exist. First, although many possible risk factors from the eligible articles were extracted, a high degree of heterogeneity was detected when analyzing the combined incidence and conducting the subgroup analyses. Second, it was not possible to analyze the incidence of PTSD among survivors after earthquakes by age, religious beliefs, nationality, social support and genetic background because these data were not reported in most of eligible articles.

## Conclusions

Results of this study suggest that nearly 1 in 4 earthquake survivors are diagnosed as having PTSD. Thus, this is remarkable evidence that natural disasters, such as earthquakes, may have a great influence on survivors’ mental health. Therefore, the local government should plan effective psychological interventions for earthquake survivors. However, there might be a considerable amount of uncertainty regarding the incidence of PTSD after earthquakes due to the high degree of heterogeneity observed in the previous studies. Thus, future studies should aim at discovering more possible risk factors for PTSD after earthquakes, especially genetic background.

## Abbreviations

PTSD, post-traumatic stress disorder; PRISMA, preferred reporting items for systematic reviews and meta-analyses; DSM-IV, diagnostic and statistical manual of mental disorders, 4th edition; SPSS, statistical package for the social sciences; 95 % CI, 95 % confidence interval; MINI, mini international neuropsychiatric interview; PCL-C, PTSD checklist-civilian version; CPTSD-RI, child–PTSD reaction index; TSSC, traumatic stress symptom checklist; HTQ, harvard trauma questionnaire; PTSD-RI, PTSD reaction index; IES-R, impact of event scale-revised; ChIPS, children’s interview for psychiatric syndromes; CPSS, child PTSD symptom scale; SCID, structured clinical interview for DSM-IV disorders; CRIES, children's revised impact of event scale; PTSD-SS, PTSD self-rating scale; DTS, Davidson trauma scale; PCL, PTSD checklist, PDS, post traumatic stress diagnostic scale

## Additional file

Additional file 1: Search strategies: details of search strategy. (DOCX 14 kb)

## References

[1] Dell'Osso L, Carmassi C, Massimetti G, Daneluzzo E, Di Tommaso S, Rossi A (2011). Full and partial PTSD among young adult survivors 10 months after the L'Aquila 2009 earthquake: Gender differences. J Affect Disord.

[2] Zhang W, Jiang X, Ho KW, Wu D (2011). The presence of post-traumatic stress disorder symptoms in adolescents three months after an 8.0 magnitude earthquake in southwest China. J Clin Nurs.

[3] Zhang W, Liu H, Jiang X, Wu D, Tian Y (2014). A longitudinal study of posttraumatic stress disorder symptoms and its relationship with coping skill and locus of control in adolescents after an earthquake in China. PLoS One.

[4] Neria Y, Nandi A, Galea S (2008). Post-traumatic stress disorder following disasters: a systematic review. Psychol Med.

[5] Zhang Z, Ran MS, Li YH, Ou GJ, Gong RR, Li RH (2012). Prevalence of post-traumatic stress disorder among adolescents after the Wenchuan earthquake in China. Psychol Med.

[6] Zhang Y, Ho SM (2011). Risk factors of posttraumatic stress disorder among survivors after the 512 wenchuan earthquake in China. PLoS One.

[7] Dell'Osso L, Carmassi C, Massimetti G, Stratta P, Riccardi I, Capanna C (2013). Age, gender and epicenter proximity effects on post-traumatic stress symptoms in L'Aquila 2009 earthquake survivors. J Affect Disord.

[8] Celebi Oncu E, Wise AM (2010). The effects of the 1999 Turkish earthquake on young children: analyzing traumatized children's completion of short stories. Child Dev.

[9] Chan CL, Wang CW, Ho AH, Qu ZY, Wang XY, Ran MS (2012). Symptoms of posttraumatic stress disorder and depression among bereaved and non-bereaved survivors following the 2008 Sichuan earthquake. J Anxiety Disord.

[10] Loney PL, Chambers LW, Bennett KJ, Roberts JG, Stratford PW (1998). Critical appraisal of the health research literature: prevalence or incidence of a health problem. Chronic Dis Can.

[11] Edmondson D, Richardson S, Fausett JK, Falzon L, Howard VJ, Kronish IM (2013). Prevalence of PTSD in Survivors of Stroke and Transient Ischemic Attack: A Meta-Analytic Review. PLoS One.

[12] Chen L, Liu A (2015). The Incidence of Posttraumatic Stress Disorder After Floods: A Meta-Analysis. Disaster Med Public Health Prep.

[13] Udomratn P (2008). Mental health and the psychosocial consequences of natural disasters in Asia. Int Rev Psychiatry.

[14] Galea S, Nandi A, Vlahov D (2005). The epidemiology of post-traumatic stress disorder after disasters. Epidemiol Rev.

[15] Ma X, Liu X, Hu X, Qiu C, Wang Y, Huang Y (2011). Risk indicators for post-traumatic stress disorder in adolescents exposed to the 5.12 Wenchuan earthquake in China. Psychiatry Res.

[16] Armenian HK, Morikawa M, Melkonian AK, Hovanesian AP, Haroutunian N, Saigh PA (2000). Loss as a determinant of PTSD in a cohort of adult survivors of the 1988 earthquake in Armenia: implications for policy. Acta Psychiatr Scand.

[17] Liu A, Tan H, Zhou J, Li S, Yang T, Wang J (2006). An epidemiologic study of posttraumatic stress disorder in flood victims in Hunan China. Can J Psychiatry.

[18] Tolin DF, Foa EB (2006). Sex differences in trauma and posttraumatic stress disorder: a quantitative review of 25 years of research. Psychol Bull.

[19] Lilly MM, Pole N, Best SR, Metzler T, Marmar CR (2009). Gender and PTSD: What can we learn from female police officers?. J Anxiety Disord.

[20] Priebe S, Grappasonni I, Mari M, Dewey M, Petrelli F, Costa A (2009). Posttraumatic stress disorder six months after an earthquake: findings from a community sample in a rural region in Italy. Soc Psychiatry Psychiatr Epidemiol.

[21] Koenen KC, Nugent NR, Amstadter AB (2008). Gene-environment interaction in posttraumatic stress disorder: review, strategy and new directions for future research. Eur Arch Psychiatry Clin Neurosci.

[22] Wu HC, Chou P, Chou FH, Su CY, Tsai KY, Ou-Yang WC (2006). Survey of quality of life and related risk factors for a Taiwanese village population 3 years post-earthquake. Aust N Z J Psychiatry.

[23] Chou FH, Su TT, Chou P, Ou-Yang WC, Lu MK, Chien IC (2005). Survey of psychiatric disorders in a Taiwanese village population six months after a major earthquake. J Formos Med Assoc.

[24] Flores EC, Carnero AM, Bayer AM (2014). Social capital and chronic post-traumatic stress disorder among survivors of the 2007 earthquake in Pisco Peru. Soc Sci Med.

[25] Kadak MT, Nasiroǧlu S, Boysan M, Aydin A (2013). Risk factors predicting posttraumatic stress reactions in adolescents after 2011 Van earthquake. Compr Psychiatry.

[26] Zhou X, Song H, Hu M, Li X, Cai Y, Huang G (2015). Risk factors of severity of post-traumatic stress disorder among survivors with physical disabilities one year after the Wenchuan earthquake. Psychiatry Res.

[27] Önder E, Tural Ü, Aker T, Kiliç C, Erdoǧan S (2006). Prevalence of psychiatric disorders three years after the 1999 earthquake in Turkey: Marmara Earthquake Survey (MES). Soc Psychiatry Psychiatr Epidemiol.

[28] Başoǧlu M, Kiliç C, Şalcioǧlu E, Livanou M (2004). Prevalence of posttraumatic stress disorder and comorbid depression in earthquake survivors in Turkey: An epidemiological study. J Trauma Stress.

[29] Zhang LP, Zhao Q, Luo ZC, Lei YX, Wang Y, Wang PX (2015). Prevalence and risk factors of posttraumatic stress disorder among survivors five years after the "Wenchuan" earthquake in China. Health Qual Life Outcomes.

[30] Kun P, Han S, Chen X, Yao L (2009). Prevalence and risk factors for posttraumatic stress disorder: A cross-sectional study among survivors of the Wenchuan 2008 earthquake in China. Depress Anxiety.

[31] Roussos A, Goenjian AK, Steinberg AM, Sotiropoulou C, Kakaki M, Kabakos C (2005). Posttraumatic stress and depressive reactions among children and adolescents after the 1999 earthquake in Ano Liosia Greece. Am J Psychiatr.

[32] Cénat JM, Derivois D (2015). Long-term outcomes among child and adolescent survivors of the 2010 Haitian earthquake. Depress Anxiety.

[33] Fu Y, Chen Y, Wang J, Tang X, He J, Jiao M (2013). Analysis of prevalence of PTSD and its influencing factors among college students after the Wenchuan earthquake. Child and Adolescent Psychiatry and Mental Health.

[34] Hsu CC, Chong MY, Yang P, Yen CF (2002). Posttraumatic stress disorder among adolescent earthquake victims in Taiwan. J Am Acad Child And Adolescent Psychiatry.

[35] Jia X, Ying L, Zhou X, Wu X, Lin C (2015). The effects of extraversion, social support on the posttraumatic stress disorder and posttraumatic growth of adolescent survivors of the Wenchuan earthquake. PLoS One.

[36] Tian Y, Wong TKS, Li J, Jiang X (2014). Posttraumatic stress disorder and its risk factors among adolescent survivors three years after an 8.0 magnitude earthquake in China. BMC Public Health.

[37] Wang R, Wang L, Li Z, Cao C, Shi Z, Zhang J (2013). Latent structure of posttraumatic stress disorder symptoms in an adolescent sample one month after an earthquake. J Adolesc.

[38] Zhang J, Zhu S, Du C, Zhang Y (2015). Posttraumatic stress disorder and somatic symptoms among child and adolescent survivors following the Lushan earthquake in China: A six-month longitudinal study. J Psychosom Res.

[39] Gokcen C, Sahingöz M, Annagür BB (2013). Does a non-destructive earthquake cause posttraumatic stress disorder? A cross-sectional study. Eur Child Adolesc Psychiatry.

[40] Chan CL, Wang CW, Qu Z, Lu BQ, Ran MS, Ho AH (2011). Posttraumatic Stress Disorder Symptoms Among Adult Survivors of the 2008 Sichuan Earthquake in China. J Trauma Stress.

[41] Fan F, Zhang Y, Yang Y, Mo L, Liu X (2011). Symptoms of posttraumatic stress disorder, depression, and anxiety among adolescents following the 2008 Wenchuan earthquake in China. J Trauma Stress.

[42] Guo J, Wu P, Tian D, Wang X, Zhang W, Zhang X (2014). Post-traumatic Stress Disorder among adult survivors of the Wenchuan Earthquake in China: A repeated cross-sectional study. J Anxiety Disord.

[43] Jia Z, Shi L, Duan G, Liu W, Pan X, Chen Y (2013). Traumatic experiences and mental health consequences among child survivors of the 2008 Sichuan earthquake: a community-based follow-up study. BMC Public Health.

[44] Ying LH, Wu XC, Lin CD, Chen C (2013). Prevalence and predictors of posttraumatic stress disorder and depressive symptoms among child survivors 1 year following the Wenchuan earthquake in China. Eur Child Adolesc Psychiatry.

[45] Xu J, Song X (2011). Posttraumatic stress disorder among survivors of the Wenchuan earthquake 1 year after: Prevalence and risk factors. Compr Psychiatry.

[46] Ali M, Farooq N, Bhatti MA, Kuroiwa C (2012). Assessment of prevalence and determinants of posttraumatic stress disorder in survivors of earthquake in Pakistan using Davidson trauma scale. J Affect Disord.

[47] Ayub M, Poongan I, Masood K, Gul H, Ali M, Farrukh A (2012). Psychological morbidity in children 18 months after Kashmir Earthquake of 2005. Child Psychiatry Hum Dev.

[48] Cénat JM, Derivois D (2014). Assessment of prevalence and determinants of posttraumatic stress disorder and depression symptoms in adults survivors of earthquake in Haiti after 30 months. J Affect Disord.

[49] Gigantesco A, Mirante N, Granchelli C, Diodati G, Cofini V, Mancini C (2013). Psychopathological chronic sequelae of the 2009 earthquake in L'Aquila, Italy. J Affect Disord.

[50] Liu ZY, Yang YF, Ye YL, Zeng ZQ, Xiang YJ, Yuan P (2010). One-year follow-up study of post-traumatic stress disorder among adolescents following the Wen-Chuan earthquake in China. Biosci Trends.

[51] Naeem F, Ayub M, Masood K, Gul H, Khalid M, Farrukh A (2011). Prevalence and psychosocial risk factors of PTSD: 18 months after Kashmir earthquake in Pakistan. J Affect Disord.

[52] Parvaresh N, Bahramnezhad A (2009). Post-traumatic stress disorder in Bam-survived students who immigrated to Kerman, four months after the earthquake. Arch Iran Med.

[53] Takeda T, Tadakawa M, Koga S, Nagase S, Yaegashi N (2013). Relationship between dysmenorrhea and posttraumatic stress disorder in Japanese high school students 9 months after the Great East Japan Earthquake. J Pediatr Adolesc Gynecol.

[54] Wang B, Ni C, Chen J, Liu X, Wang A, Shao Z (2011). Posttraumatic stress disorder 1 month after 2008 earthquake in China: Wenchuan earthquake survey. Psychiatry Res.

[55] Wang L, Cao C, Wang R, Qing Y, Zhang J, Zhang XY (2013). PAC1 receptor (ADCYAP1R1) genotype is associated with PTSD's emotional numbing symptoms in Chinese earthquake survivors. J Affect Disord.

[56] Wang W, Fu W, Wu J, Ma XC, Sun XL, Huang Y (2012). Prevalence of PTSD and depression among junior middle school students in a rural town far from the epicenter of the Wenchuan Earthquake in China. PLoS One.

[57] Wen J, Shi YK, Li YP, Yuan P, Wang F (2012). Quality of life, physical diseases, and psychological impairment among survivors 3 years after Wenchuan earthquake: A population based survey. PLoS One.

[58] Zhang Z, Shi Z, Wang L, Liu M (2011). One year later: Mental health problems among survivors in hard-hit areas of the Wenchuan earthquake. Public Health.

[59] Zhang Z, Wang W, Shi Z, Wang L, Zhang J (2012). Mental Health Problems among the Survivors in the Hard-Hit Areas of the Yushu Earthquake. PLoS One.

[60] Hou FS, Li T, Li J, Hu XQ, Liu ZY, Yuan P (2011). The effects of demographic features on differences in sensitivity between PCL-C and SCL-90 scores in a follow-up study in secondary school students in the Wenchuan earthquake region. Biomed Environ Sci.

[61] Lau JT, Yu X, Zhang J, Mak WW, Choi KC, Lui WW (2010). Psychological distress among adolescents in Chengdu, Sichuan at 1 month after the 2008 Sichuan earthquake. J Urban Health.

[62] Liu X, Yang Y, Yuan P, Zhang X, Han Y, Cao Y (2010). A study of the relationship between mental health and menstrual abnormalities in female middle school students from postearthquake Wenchuan. Biosci Trends.

[63] Ying L, Wu X, Lin C, Jiang L (2014). Traumatic severity and trait resilience as predictors of posttraumatic stress disorder and depressive symptoms among adolescent survivors of the Wenchuan earthquake. PLoS One.

[64] Kun P, Tong X, Liu Y, Pei X, Luo H (2013). What are the determinants of post-traumatic stress disorder: Age, gender, ethnicity or other? Evidence from 2008 Wenchuan earthquake. Public Health.

[65] Sezgin U, Punamäki RL (2012). Earthquake trauma and causal explanation associating with PTSD and other psychiatric disorders among South East Anatolian women. J Affect Disord.

