# Supplementary material for: The incidence of post-traumatic stress disorder among survivors after earthquakes:a systematic review and meta-analysis
Source: BMC Psychiatry. 2016 Jun 7;16:188. doi: 10.1186/s12888-016-0891-9 (PMC4895994; doi:10.1186/s12888-016-0891-9)
Supplement: Additional file 1: — Search strategies: details of search strategy. (DOCX 14 kb) [file 12888_2016_891_MOESM1_ESM.docx]

**Search strategies**

The search terms for PsycARTICLES were: (AB PTSD OR AB posttraumatic stress disorder OR AB post traumatic stress disorder) AND (AB earthquake or AB earthquakes);

The search terms for Embase were: ('post traumatic stress disorder':ab,ti OR 'posttraumatic stress disorder':ab,ti OR 'PTSD':ab,ti) AND ('earthquake':ab,ti' OR earthquakes':ab,ti);

The search terms for Web of Science were: TS=Earthquakes AND TS=Stress Disorders, Post-Traumatic;

The search terms for PubMed were: ("Earthquakes"[Mesh]) AND "Stress Disorders, Post-Traumatic"[Mesh].
